# Biobanks in veterinary forensic medicine: A systematic review on Advances, challenges, and applications in combating wildlife trafficking

**DOI:** 10.14202/vetworld.2026.933-947

**Published:** 2026-03-12

**Authors:** Natália Freitas de Souza, Teng Fwu Shing, Leticia Gondim Souto, Fernanda de Freitas Alves Vieira, Juliana Keiko Louriçal Firmo Nishihara, Nadia Yumi Yamamoto Dos Santos, Bianca Parcianello Rostirolla, Marcela da Costa Gomes, Fernanda Barthelson Carvalho de Moura, Noeme Sousa Rocha

**Affiliations:** Department of Veterinary Clinic, School of Veterinary Medicine and Animal Science, São Paulo State University – UNESP, Botucatu, Brazil

**Keywords:** artificial intelligence, biodiversity conservation, biobank, conservation genetics, DNA barcoding, forensic applications, genetic resource preservation, illegal trade monitoring, One Health, species identification, veterinary forensic medicine, wildlife screening centers, wildlife trafficking

## Abstract

**Background and Aim:**

Biobanks represent organized repositories of biological samples linked to associated data, designed for long-term scientific, clinical, and forensic utilization. In veterinary medicine, animal biobanks facilitate biomedical research, genetic resource preservation, species conservation, and forensic investigations. The present systematic review aimed to synthesize advances, persistent challenges, and practical applications of biobanks in veterinary forensic medicine, with particular emphasis on their contribution to detection, investigation, and suppression of wildlife trafficking.

**Materials and Methods:**

A systematic literature search was conducted across Periódicos Capes, PubMed, SciELO, and ScienceDirect databases, covering publications from 2013 to 2023. Search strings combined terms such as “animal biobank”, “animal biorepository”, “wildlife forensic”, “wildlife trafficking”, and “forensic veterinary” (English), together with Portuguese equivalents. Only peer-reviewed articles published in English or Portuguese that explicitly addressed biobanks in veterinary forensic contexts or wildlife crime were included. The review adhered to PRISMA 2020 guidelines. Screening involved title/abstract evaluation followed by full-text assessment. Data were narratively synthesized.

**Results:**

Of 1,495 records identified, 15 studies fulfilled all inclusion criteria after exclusion of 1,460 irrelevant or non-qualifying publications. No eligible articles appeared between 2013 and 2014. From 2015 onward, publications demonstrated progressive refinement, transitioning from molecular barcoding for species identification toward integrated applications in geographic origin assignment, chain-of-custody documentation, and evidentiary support in judicial proceedings. Key materials included DNA from muscle, scales, claws, and feathers; cryopreserved gonadal tissues; and somatic cells derived from minimally invasive sources (e.g., feather follicles) or roadkill specimens. Studies highlighted particular utility in identifying fraudulently labeled fishery products, counterfeit mammalian derivatives (e.g., fake tiger claws), and confiscated pangolin scales, as well as in tracing trafficking routes in high biodiversity regions.

**Conclusion:**

Veterinary forensic biobanks offer substantial potential for accurate species and geographic provenance determination, thereby strengthening enforcement against illegal wildlife trade. Nevertheless, implementation remains constrained by absent standardized operating procedures, limited practitioner awareness, fragmented reference databases, inadequate inter-institutional connectivity, and elevated logistic/financial demands. Regionalized biobanks integrated with wildlife screening centers (CETAS), harmonized chain-of-custody protocols, and artificial intelligence-supported data curation are proposed as priority strategies to translate existing scientific advances into routine forensic and conservation practice.

## INTRODUCTION

In recent decades, biobanks and biorepositories have emerged as pivotal resources in advancing both human and veterinary medical research, representing a cornerstone of the precision medicine paradigm. The Organization for Economic Cooperation and Development defines a biobank as an organized collection of biological material primarily intended for scientific and academic research, linked to associated information databases and governed by stringent operational protocols. Such repositories support multiple investigations and are designed for sustained utilization over years or decades. Originally applied to human-derived samples, the term “biobank” has since been extended to encompass biological specimens of any origin, including nonhuman sources [1-3].

A biorepository is frequently employed synonymously with a biobank owing to overlapping conceptual features. Nevertheless, a biorepository typically serves a defined research project or objective and is often temporary in nature, whereas a biobank is structured for long-term preservation and broad scientific application [1–3].

Biobanks exhibit considerable diversity in purpose and scope. They may store neoplastic cells, germ cells, DNA/RNA, biofluids, proteins, or subcellular constituents, thereby mirroring the progressive expansion of knowledge in health-related disciplines. Irrespective of the specific category of biological material preserved, linkage to comprehensive clinical or phenotypic metadata constitutes an indispensable requirement for ensuring sample traceability [3]. To achieve national and international recognition and utility, collected specimens must adhere to rigorously standardized operating procedures, with all processes thoroughly documented [3]. Internationally, ISO 20387:2018 (Biobanking, General requirements for biobanking) provides the principal normative framework for sample validation. In Brazil, this has been adapted as ABNT NBR ISO 20387:2020 (Biotechnology, Biobanking activities, General requirements for biobanking activities), harmonizing international standards with national requirements [3–5].

In veterinary medicine, the establishment of the earliest biobanks dates to 2006, initially focused on biopreservation, biodiversity conservation, infectious disease research, and genetic studies. Over subsequent years, biobanking has attained increasing prominence, demonstrating critical relevance not only for comparative biomedical investigations but also for elucidating the interconnected health dynamics among animals, humans, and ecosystems [6, 7]. Although numerous animal biobanks currently operate globally, the majority primarily facilitate human-oriented comparative research. Concurrently, dedicated institutional initiatives have emerged to conserve biological material across diverse nonhuman taxa for purposes encompassing species preservation, genetic resource safeguarding, and pathogen characterization [7].

Biobanks fulfill a particularly vital function in the conservation of threatened and endangered species through the long-term storage of DNA, tissue specimens, blood, bone marrow, embryos, and oocytes. Ex situ reproductive programs rely heavily on such preserved material to sustain viable population sizes, preserve genetic diversity, and enable detailed investigations of physiology and behavior [8, 9]. Moreover, recent evaluations have explored the utility of virtual biobanks for animal-derived samples, integrating derived data with epidemiological surveillance metrics and facilitating pathogen identification and genomic sequencing for ongoing infectious disease monitoring [10].

As in human biobanking, rigorous adherence to standardized operating procedures, together with strict compliance with ethical and regulatory frameworks, remains essential. Parallel advancement between veterinary and human biobanking domains is therefore imperative. Institutions functioning as biobanks, including zoological collections, academic centers, agricultural enterprises, research facilities, and veterinary hospitals, must establish harmonized guidelines governing sample collection, preservation, storage, and data sharing [3, 6]. Within the legal domain, veterinarians assume multifaceted responsibilities in wildlife-related cases, participating in integrated networks that encompass regulatory authorities, criminal and civil legislation, and forensic diagnostic services. Wildlife specimens may serve as either victims or commodities in trafficking networks, which frequently intersect with organized crime involving narcotics, firearms, tax evasion, and other illicit financial activities [11, 12].

The global wildlife trafficking economy, encompassing live animals and derived products, constitutes a well-documented transnational criminal enterprise. Escalating threats of species extinction, disruption of ecological equilibrium, and heightened risk of emerging and re-emerging zoonotic pathogens underscore the urgent necessity for robust preventive and enforcement strategies. In this context, deployment of cutting-edge technologies and sustained conservation measures, including the strategic development and maintenance of biobanks for both forensic and biodiversity preservation objectives, assumes paramount importance [13]. Reliable global estimates of illegally traded wildlife volumes remain limited, largely attributable to challenges in accurate species identification and the paucity of robust reference databases. It is estimated that approximately 70% of wildlife trafficking incidents evade official documentation owing to inadequate taxonomic determination of specimens or their derivatives [13, 14].

Although biobanks have demonstrated substantial value in human forensic applications and comparative veterinary studies, a notable deficiency persists in systematic evaluations of their targeted deployment within veterinary forensic medicine, especially concerning wildlife criminality. Predominant scholarly focus has centered on biobanking for conservation genetics, assisted reproductive technologies, and infectious disease monitoring, with forensic assimilation remaining disjointed and inadequately examined [1–10]. For example, while DNA barcoding and related molecular methodologies have facilitated species delineation in discrete forensic scenarios [15–19], the lack of integrative syntheses connecting these approaches to biobank frameworks impedes the formulation of uniform forensic guidelines. Furthermore, impediments including erratic sample provenance documentation, constrained cross-institutional data interoperability, and insufficient recognition among forensic specialists perpetuate the underreporting of wildlife trafficking events, which globally approximate 70% due to identification deficiencies [13, 14]. This shortfall is especially pronounced in regions of elevated biodiversity, such as Brazil, where frequent confiscations of erroneously labeled animal commodities highlight the imperative for cohesive biobanking approaches to bolster evidential integrity and regulatory effectiveness [12, 16]. Notably, prior literature lacks a dedicated systematic appraisal that traces the trajectory of biobanks from conservation instruments to forensic resources, nor does it advance pragmatic models for surmounting operational, normative, and infrastructural hurdles in mitigating illicit wildlife commerce.

This systematic review endeavors to elucidate the progressions, enduring obstacles, and operational utilities of biobanks within veterinary forensic medicine, placing particular accentuation on their contributions to thwarting wildlife trafficking. In particular, it aspires to: (i) appraise the chronological evolution of biobank-centric investigations spanning 2013 to 2023, underscoring transitions toward forensic genomic applications and geo-spatial provenance determination; (ii) delineate principal constraints, encompassing the dearth of unified procedural norms, referential repositories, and evidentiary chain-of-custody benchmarks; (iii) advocate deployment frameworks, including decentralized biobanks interfaced with wildlife triage facilities and artificial intelligence-enhanced data stewardship; and (iv) emphasize the forensic capabilities for taxonomic verification, source localization, and courtroom admissibility in trafficking inquiries. Through the amalgamation of data across varied taxonomic groups and international settings, this investigation aims to furnish an foundational schema for stakeholders in policy, veterinary practice, and conservation to actualize biobanks as versatile instruments in safeguarding biodiversity and advancing One Health paradigms.

## MATERIALS AND METHODS

### Ethical approval

**Figure 1:**
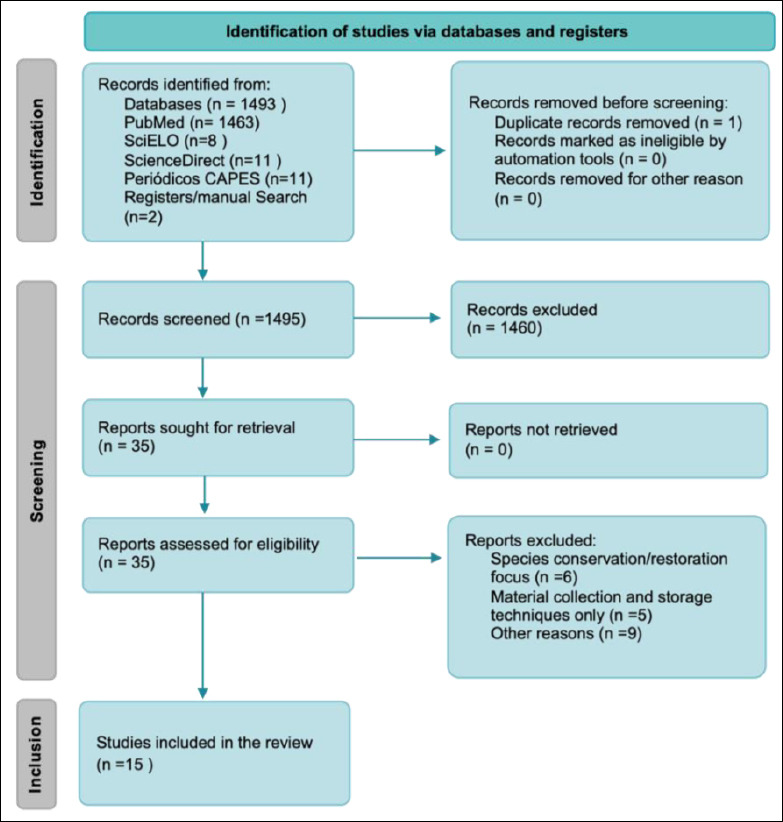
PRISMA 2020 flowchart. Adapted in accordance with PRISMA 2020 guidelines, illustrating the stages of identification, screening, eligibility assessment, and inclusion of studies in the systematic review. Image generated with assistance from ChatGPT 5.2.

This investigation adopted a descriptive and retrospective design, encompassing peer-reviewed scientific publications pertinent to the utilization of biobanks in veterinary forensic medicine, with particular emphasis on wildlife-related applications. The review protocol was not prospectively registered in PROSPERO. The study design adhered to an adapted version of the Preferred Reporting Items for Systematic Reviews and Meta-Analyses (PRISMA 2020) guidelines (Figure 1).

The research proposal was formally registered with São Paulo State University (UNESP) under registration number 000.069. The protocol conformed to the directives of the National Council for the Control of Animal Experimentation (CONCEA) and received approval from the Animal Use Ethics Committee of the Faculty of Veterinary Medicine and Animal Science (FMVZ). No live animal experimentation was conducted, as the study relied exclusively on publicly available, previously published scientific literature. Consequently, no additional ethical clearance for animal involvement was required. All data extracted and analyzed were derived from openly accessible sources.

### Study period and location

The systematic literature search was conducted from 2013 to 2023 across Periódicos Capes, PubMed, SciELO, and ScienceDirect databases, covering publications.

### Information sources and search strategies

Literature retrieval spanned publications issued between 2013 and 2023, encompassing articles written in English and Portuguese. The following electronic databases were systematically queried: Periódicos Capes, PubMed, SciELO, and ScienceDirect. The search process occurred between 2023 and 2024.

The Boolean operator AND was employed to combine search terms, while quotation marks were utilized to ensure phrase-specific retrieval. English-language keywords included “animal biobank,” “animal biorepository,” “wildlife forensic,” “wildlife trafficking,” and “forensic veterinary.” Corresponding Portuguese terms comprised “biobanco animal,” “biorrepositório animal,” “tráfico de animais silvestres,” “medicina veterinária forense,” and “medicina legal veterinária.”

Individual terms (“animal biobank,” “animal biorepository,” “biobanco animal,” “biorrepositório animal”) and their variations were searched independently and in pairwise combination with the remaining keywords within each respective language, always linked via the AND operator, to maximize specificity. Database-specific syntax adjustments were applied as necessary.

The initial search yielded 1495 records. Following removal of duplicates and irrelevant material, 1460 records were excluded, resulting in 35 potentially eligible publications. After detailed full-text screening against predefined eligibility criteria, 15 studies were ultimately included in the qualitative synthesis.

The search strategy deliberately avoided restriction to any single species or taxonomic group. Instead, the scope encompassed biobanks addressing wildlife broadly (including mammals, reptiles, birds, fish, and amphibians), reflecting the predominantly non-taxonomically restricted nature of the retrieved literature.

### Inclusion and exclusion criteria

Manuscripts were considered eligible if they met the following criteria: full-text articles published in English or Portuguese; available online; presenting robust, evidence-based findings; and explicitly addressing the application of biobanks within veterinary forensic medicine or wildlife crime contexts.

Exclusion criteria encompassed duplicate publications, incomplete manuscripts, conference abstracts (simple or extended), posters, book chapters, theses, technical reports, conference proceedings, and studies focused exclusively on species conservation or restoration without forensic relevance.

To augment comprehensiveness, a supplementary manual search was performed by scrutinizing the reference lists of all included and eligible articles. Limitations and future research directions identified in the primary studies were also extracted and synthesized.

### Selection of publications

Article screening proceeded in sequential stages. Initial evaluation involved title assessment for topical relevance. Abstracts of retained records were then reviewed to ascertain scientific pertinence and alignment with the core research terms. Full texts of preliminarily included articles were subsequently examined in detail. During full-text appraisal, emphasis was placed on documenting the biobank type, stated objectives, biological materials preserved, species or taxonomic groups involved, reported limitations, and articulated future perspectives.

### Data extraction and management

Data extraction was performed independently by a single reviewer utilizing a structured tabular format created in Microsoft Word 365. Extracted variables comprised publication reference, year of publication, principal thematic focus (derived from title and content), journal name, and Digital Object Identifier (DOI) where available. The collated data underwent narrative synthesis to integrate findings across the included studies.

### Quality assessment

Quality evaluation was conducted collaboratively by the co-authors in conjunction with independent external researchers. Critical appraisal focused on the clarity and novelty of the central research question, methodological rigor, scientific validity of presented data, overall relevance to the field, and prospective translational or practical applicability.

Principal limitations of the present review include the restricted volume of publications addressing this niche topic, the fragmented and heterogeneous nature of existing evidence, and the limited direct linkage between reported findings and routine applicability in wildlife trafficking investigations. Consequently, this work serves primarily as a foundational proposal to bridge current scientific knowledge with practical enforcement interventions.

Strengths of the review lie in its systematic consolidation and reappraisal of an under-represented subject, thereby elevating its visibility within both academic and applied domains. This consolidation holds particular relevance amid the persistent global prevalence of wildlife trafficking, where increasingly sophisticated, evidence-informed countermeasures are urgently required.

## RESULTS

### Characteristics of the included studies

**Figure 2:**
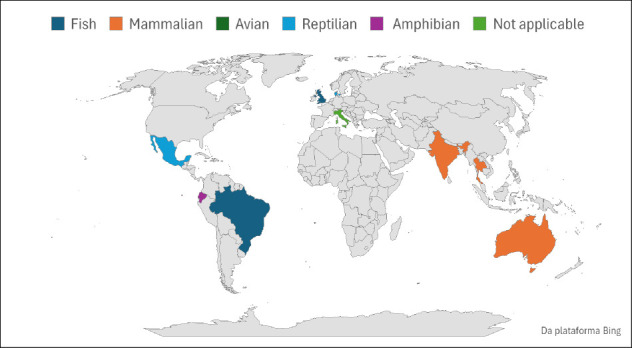
Global distribution of biobanks according to animal group. The map depicts the geographic location of biobanks or related studies

**Figure 3:**
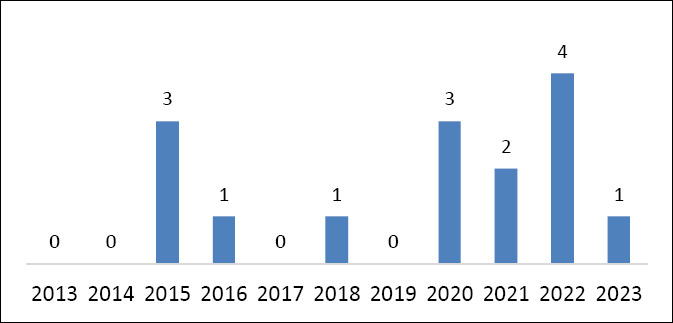
Annual number of publications related to wildlife biobanks (2013–2023).

**Figure 4:**
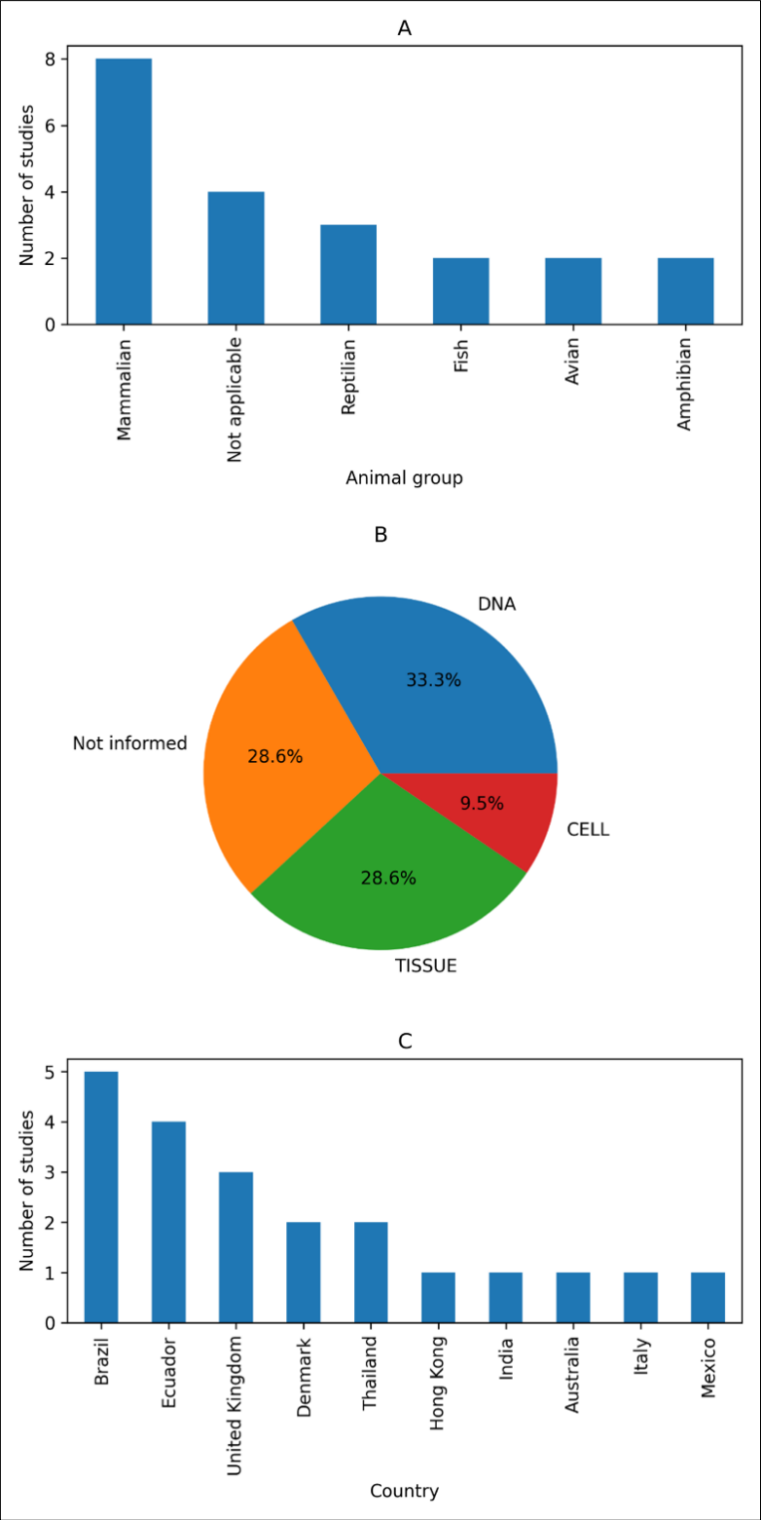
Global distribution of biobanks stratified by animal group, sample storage type, and number of studies per country. Composite visualization integrating geographic distribution, taxonomic focus (fish, mammals, avians, reptiles, amphibians, not applicable), and storage modalities across reporting countries. Image generated with assistance from ChatGPT 5.2.

**Figure 5:**
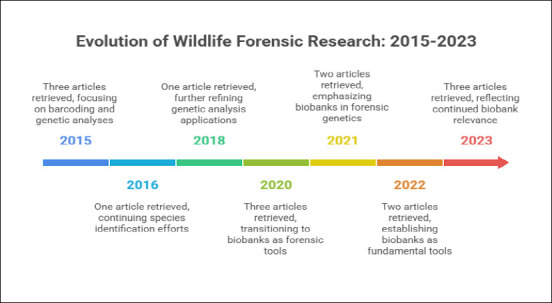
Evolution of wildlife forensic research: flow diagram overview of the 15 selected publications. Diagram summarizes the annual distribution and primary thematic focus of included studies from 2013 to 2023, highlighting progression in research emphasis over time. Image created with assistance from NapkinAI.

In total, 1495 research papers were identified. Of these, 1460 were excluded, 35 studies were considered eligible for the present review. However, after screening, only 15 papers were included in this systematic review as they met all the criteria for this research. In accordance with the selection criteria applied in this study, no related articles were retrieved between 2013 and 2014. However, between 2015 and 2023, 15 articles were recovered. From 2015 onward, there was a refinement in publications related to the topic, with three articles retrieved in 2015, one each in 2016 and 2018, three each in 2020 and 2023, and two each in 2021 and 2022 (Figures 1 and 2; Table 1). There has been a refinement in the approaches and focuses of the published research, despite no increase in the number of publications. Between 2015 and 2018, the articles focused on specific applications of barcoding and genetic analyses aimed at species identification, tracking, and enforcement of illegal products. From 2020 onward, biobanks served as a complementary tool to forensic genetics, gaining relevance in the connection between conservation, justice, and public health. In the most recent years, i.e., 2022 and 2023, biobanks have become established as fundamental tools, with practical applications directed toward genetic preservation and technical-scientific support for criminal investigations involving wildlife (Figures 3–5). This evolution reflects a broadening of approaches, with increased interdisciplinarity and greater emphasis on the systematic collection of samples, including alternative sources, such as animals that died on roadways. This technical-scientific advancement occurs alongside growing social concern for animal-related issues, reflecting a change in public values, particularly in the forensic context. Greater access to and dissemination of information on animal welfare, through documentaries, news, and campaigns, has contributed to a cultural redirection in society, fostering a shift in mindset and evolving perspectives toward animals [11].

**Table 1:** Characteristics of the included studies.

Author	Country	Species/animal group	Biological material	Forensic application	Main findings
Carvalho *et al.* (2015)	Brazil	Fish species	DNA (muscle sample)	Identifying seafood fraud using DNA barcodes and monitoring fisheries and illegal trade	Useful for identifying commercialization of illegal species; identification of endangered species and illegal mislabeling.
Ogden L (2015)	United Kingdom	Fish; elephant	DNA from the ivory sample	Using DNA samples to identify a specific population or geographical location, species identification	In conjunction with other techniques, DNA can be used to assist law enforcement in identifying geographic origin.
Zhang *et al.* (2015)	Hong Kong	Pangolin	Mitochondrial DNA (scale sample)	Molecular tracing of pangolin scales confiscated for conservation and illegal trade monitoring in Southeast Asia	This study illustrates the value of applying DNA forensics to the monitoring of illegal wildlife trade.
Sharma *et al.* (2016)	India	Tiger	DNA (claw sample)	Pioneer identification of fake tiger claws in India using morphometric and DNA-based analysis in wildlife forensics	“A morphometric and genetic reference database should be developed to identify species in the illegal wildlife trade. Morphological features and DNA profiles need to be used for better implementation of the Wildlife (Protection) Act, 1972 of India and other laws/treaties in Southeast Asia.”
Karmacharya *et al*. (2018)	Australia	Tiger	Nuclear DNA microsatellite genotype and sex profiles and geo-spatial information	Species, sex, and geolocation identification of seized parts of a tiger (*Panthera tigris tigris*) in Nepal: a molecular forensic approach	This study demonstrated the feasibility of using molecular forensic evidence to incriminate offenders in court in combating wildlife crime.
Cardoso *et al.* (2020)	Brazil	Birds	Feather follicle cells	Somatic feather follicle cell culture of the *Gallus domesticus* species for creating a genetic resource bank for wild birds	The extraction of somatic cells from the follicles of birds’ feathers is an excellent, viable, and efficient method for obtaining biological material for the development of biobanks.
Strand *et al.* (2020)	Denmark	Amphibia and Reptilia	Review	Biobanking for amphibian and reptile conservation and management: opportunities and challenges	Advanced research focusing on fibroblast cell lines cultured with appropriate pluripotent stem cells and advanced reproductive technologies will be an invaluable resource for species conservation and management.
Zucca *et al.* (2020)	Italy	Not applicable	Hypothesis and theory	The "Bio-Crime Model" of cross-border cooperation among veterinary public health, justice, law enforcement, and customs to tackle illegal animal trade/bio-terrorism and prevent the spread of zoonotic diseases among human population	The Bio-crime cross-border cooperation model replicates collaboration among veterinary public health, justice, and law enforcement across countries through International Police and Customs Cooperation Centers and is recognized as a European best practice due to its scalability and cost-neutral implementation.
Camacho-Sánchez *et al.* (2021)	Mexico	Sea turtle	COI DNA barcodes	DNA barcode analysis of the endangered green turtle (*Chelonia mydas*) in Mexico	DNA barcodes help assess the diversity of populations.
Perez-Espona, Consortium (2021)	United Kingdom	Not applicable	Not applicable	Conservation-focused biobanks: a valuable resource for wildlife DNA forensics	The collaboration between wildlife forensic geneticists and conservation-oriented biobanks, along with the sharing of digital DNA data within the wildlife forensics community, is needed to address the limitations in sample and data acquisition and to accelerate the effective implementation of law enforcement against wildlife crime.
Cruz *et al.* (2022)	Brazil	Not applicable	Not applicable	Biobanks of wild animals: literature review	To safeguard species profiles that are at risk, a fundamental scientific database is created using biological samples, including DNA, somatic cells, tissues, blood, germplasm, and embryos.
Santos and Silva (2022)	Brazil	Not applicable	Not applicable	Biobancos para a conservação da vida silvestre: desafios e perspectivas	Biobanks are indispensable tools for safeguarding and coordinating information on wildlife, thereby enabling a data network for developing strategies to conserve specimens with rare or endangered genotypes.
Silva *et al.* (2022)	Brazil	Collared peccaries, agoutis, cavies, and armadillos	Gonadal tissues	Biobanking and use of gonadal tissues: a promising strategy for conserving wildlife in the Caatinga biome	After *in vitro* culture or grafting, gonadal tissues can serve as sources of mature gametes for assisted reproduction and for studying gametogenesis and infertility mechanisms, highlighting the importance of species-specific protocols for tissue recovery, cryopreservation, and culture.
Sukparangsi *et al*. (2022)	Thailand	Fishing cat	Cell and tissue samples	Fishing cat cell biobanking for sustainable conservation	Tissue collection and culture procedures for zoological research facilitate cell preservation from both postmortem and living felids.
Coba-Males *et al.* (2023)	Ecuador	Classes of Amphibia, Reptilia, Aves, and Mammalia	Tissue samples	From roads to biobanks: roadkill animals as a valuable source of genetic data	Biobanks may help elucidate the structure and composition of wildlife, including the bacteria and microparasites associated with wildlife, which may pose risks to human health.

*Manual inclusion.

### Another biobank application

The earliest studies focused on molecular biology, which is not a new technique and has already been employed in courtrooms. It is an important tool not only for animal identification but also for comparative purposes and for obtaining geographic references of wildlife species. Importantly, many reference materials were originally intended for research and often lack detailed information and documentation, making their use in criminal investigations unfeasible. Additionally, there are repositories containing materials with high forensic potential; however, their existence is either unknown or inaccessible, as most professionals are unaware of their applicability in the criminal context [15]. At the national level, Brazil’s high biodiversity, particularly in the Amazon region, is a major attraction for the wildlife trafficking market. Data from the past five years indicate that wildlife-derived products are among the most frequently seized items [12].

**Figure 6:**
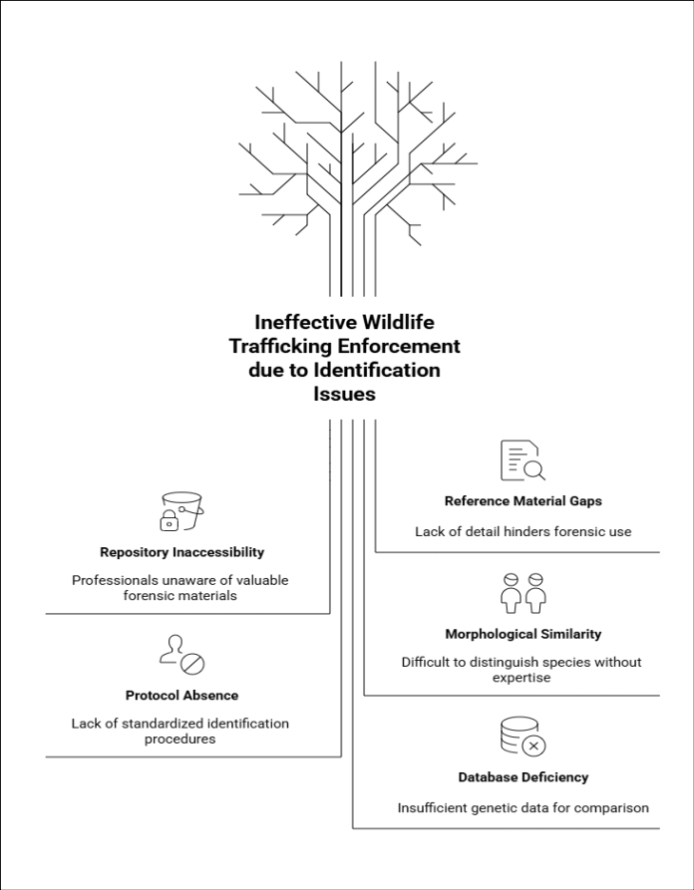
Enforcement of wildlife trafficking as an application of biobanks. Schematic representation illustrating the integration of biobank-derived data (species identification, geographic origin tracing, genetic profiling) into forensic investigative and judicial processes aimed at combating illegal wildlife trade. Image created with assistance from NapkinAI.

On this basis, one of the main examples of improper identification involves fish and their products, which may lose the essential morphological characteristics needed for species identification, such as in the case of fish filets and preprocessed seafood. These conditions can lead to species misidentification and consequently compromise forensic analysis. A previous study reported that in fish and other fishery products sold in southern and southeastern Brazil, many low-value species were marketed as high-value ones, with differences being imperceptible without in-depth genetic analysis (Figure 3–6) [16]. This is also observed not only in fish but also in pangolins and their byproducts, which are sought after for their meat and, more notably, for their scales, used in traditional Chinese medicine and to supply the illegal market. Similar issues occur with counterfeit mammal claws identified through morphometric analysis, imaging techniques, and DNA testing. The authors determined that the seized claws analyzed were derived from keratinous material of Bos taurus, which had been modified to resemble tiger or leopard claws (Figure 3–6) [17, 18]. The comparative analysis approach can be extended to other animal-derived materials, such as horns and teeth, and to additional species, and may be applied in the routine activities of agencies on the front lines of wildlife trafficking enforcement. Accurate identification remains a major challenge in most seizures due to morphological similarities among animals, the lack of standardized protocols, and the absence of a sufficiently diverse database. Morphological classification is a fundamental method for the taxonomic identification of biological materials and is a reliable and cost-effective method for identifying items in forensic analyses, provided that sufficient diagnostic characteristics are present and adequate expertise and reference materials for comparison are available. DNA analysis has become the method of choice for identification in the absence of such information [11, 19].

Another application of biobanks in routine practice is spatial georeferencing and the potential determination of specimen origins [20]. The methodology employed by researchers could be adapted for use with other animal species across regions, thereby contributing to biodiversity monitoring and endangered species protection. Implementing this approach on a global scale could strengthen the fight against illegal trafficking by supporting the formulation of public policies and more effective enforcement actions, enabling more precise targeting of trafficking routes as they develop and predicting potential new routes (Figures 3–6). For avians, studies indicate the viability of cultured cells obtained from feathers under laboratory conditions, employing a minimally invasive and promising approach. In Brazil, as well as in other countries, genetic information on birds is lacking, which is one of the limiting factors for the advancement of research in this area and, consequently, for progress in forensic applications [21]. This technique can be particularly useful in the context of wildlife trafficking, as only feathers are often recovered. These feathers are found in the environment and are naturally shed by animals. As with birds, reptile and amphibian biobanks remain scarce. Although organizations are dedicated to preserving the genetic material of these species, sample collection protocols, conservation methods, and the dissemination and accessibility of data still require improvement. The literature reports progress and challenges in reptile and amphibian biobanking, including the use of cryopreservation techniques, which may serve as models for establishing future biobanks [22]. In Brazil, studies have been conducted across regions and species groups to explore the potential forensic applications of animal biobanks [16, 21, 23]. This type of research is essential for enhancing surveillance efforts and combating wildlife trafficking, especially in the North, Northeast, and Central-West Regions, which have historically been the most affected by trafficking networks because of their high biodiversity and relatively less effective enforcement than other parts of the country [24].

### Use perspective and limitations

Although considerable information regarding wildlife biobanks is available, their implementation and effective use still present significant challenges. The validation of molecular techniques, particularly those involving DNA analysis, which require reliable, species-specific reference datasets, is a major obstacle. Additionally, the extensive taxonomic diversity of wildlife, mammals, reptiles, birds, fish, and amphibians, needs distinct methodological approaches tailored to each group’s biological particularities. Therefore, biobanks must either focus on a specific group or be equipped with specialized infrastructure capable of accommodating the unique storage and processing requirements of different taxa. This is crucial because tissue collection, preservation, and long-term storage protocols are not universally applicable across species [13, 25]. Another critical aspect to be considered is that biobanks must accurately reflect the genetic and adaptive changes that species undergo over time.

Given the accelerating pace of anthropogenic environmental changes, which drive rapid evolutionary responses, it is essential to preserve genetic material that faithfully represents organisms within their dynamic genetic and ecological contexts. This requires continuous updating and strategic sampling to maintain the relevance and scientific utility of biobanked samples in both conservation and forensic applications [26]. The logistics of transporting and preserving biological samples in biobanks, particularly with respect to the risk of pathogen transmission, require special attention. This concern becomes even more critical when storage involves the shared use of liquid nitrogen vapor, which can facilitate cross-contamination. In addition, some biological materials require specific permits for transportation, such as those issued by the Convention on International Trade in Endangered Species and must comply with strict transport and storage conditions to ensure high biosafety standards [8]. In parallel, an increasing number of biological samples are added to biobanks distributed globally each year. However, the lack of efficient computerized systems limits researchers, research centers, and other institutions’ access to these data, hindering their integration. This deficiency undermines connectivity and information sharing, thereby reducing both the potential for collaboration and the practical applicability of the stored samples [9]. The limitations and challenges related to the use of biobanks are even more complex in the context of veterinary forensic medicine, as the lack of awareness regarding the existence and potential of biobanks represents a significant barrier to their adoption. Furthermore, it is essential to ensure the integrity of the samples and associated data, which must be subjected to rigorous protocols to guarantee their validity and prevent nullification [11–20]. Maintaining an unbroken chain-of-custody for the samples is another critical challenge, ensuring that no contamination or tampering occurs, thereby ensuring their admissibility. The absence of standardized protocols and the diversity of existing species further complicate the implementation of such security measures. Moreover, the lack of specific regulations regarding biobanks in veterinary forensic medicine may lead to significant discrepancies in the use of these samples, compromising their reliability [11–20]. Furthermore, the lack of infrastructure and qualified personnel to properly manage samples in veterinary biobanks can hinder the rapid and efficient access to and sharing of information. The continuous updating of preservation techniques and the integration of different biobanks, both at the national and international levels, present challenges that must be overcome to enable the effective application of this tool in veterinary forensic medicine [1–3]. It is important to highlight that the costs of creating, implementing, and maintaining a biobank are not fully definable, which is a crucial aspect that must be explored. A strategy used by big corporations is self-financial strategies that are used to recover the costs of the biobank. However, in some cases, financial support from the government or other institutions is necessary [3]. Despite several challenges in the implementation of biobanks, advances in veterinary medicine, particularly in the context of wildlife, are evident. Although biobanks are primarily aimed at species conservation and preservation, they hold significant potential to assist in legal matters. These samples contribute to the biobank’s data composition and can be used for comparative purposes in investigations related to illegal wildlife trafficking. The morphological identification difficulties of certain species persist in many countries, particularly with respect to exotic species [13, 27, 28]. Given this limitation, the establishment of biological material databases and repositories is essential, as they provide crucial information for animal identification and enable their application on a global scale in enforcing relevant legislation. These biobanks hold significant potential as support tools in combating wildlife crimes, particularly in addressing international wildlife trafficking, thereby advancing forensic investigations [28–30]. Importantly, the biobank represents only one link in a large and complex chain, requiring multidisciplinary teams for standardization, sample collection and management, data sharing, and use for continuous improvement and refinement. There is a need to expand knowledge regarding wildlife, ecology, biological behavior, reproduction, veterinary care, and forensic aspects. In addition to veterinarians, environmental agencies and the justice system would benefit from this information, including improved enforcement of relevant legislation and proper characterization of crimes involving wildlife [9, 28, 29].

### Proposal for the implementation and use of biobanks for combating wildlife trafficking

**Figure 7:**
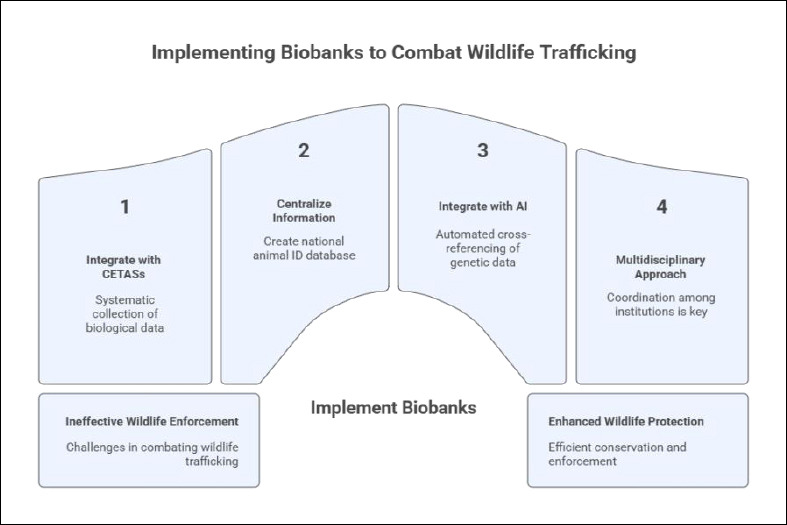
Proposed flowchart for the implementation and utilization of biobanks in combating wildlife trafficking. Stepwise diagram outlining recommended strategies, including integration with wildlife screening centers (CETAS), development of national identification databases, artificial intelligence-supported data analysis, multidisciplinary collaboration, and alignment with One Health principles. Image created with assistance from NapkinAI.

Considering Brazil’s continental dimensions, rich biodiversity, and challenges in effectively combating wildlife trafficking, the implementation of biobanks could serve as a complementary strategy to existing animal enforcement measures. Regional and national initiatives would feed collaborative networks, enhancing the efficiency of conservation and enforcement actions and contributing to wildlife protection. In this context, the implementation and utilization of these resources should be explored. One of the primary ways to implement biobanks is linked to wildlife screening centers (CETASs), which are responsible for receiving, screening, monitoring, and, when feasible, rehabilitating and releasing rescued, seized, or voluntarily surrendered animals. CETAS represents a strategic infrastructure for the systematic collection and storage of biological and morphometric data with units distributed across different regions of the country and operating under the Brazilian Institute of Environment and Renewable Natural Resources (IBAMA). The regionalization of these data would contribute to the accurate identification of samples, determination of potential areas of origin, and more effective monitoring of trafficking routes and targeted species [30]. Additionally, centralizing this information would enable the creation of a national animal identification database with a special focus on priority and endangered species. Such a database would serve as a valuable tool for the integrated action of environmental enforcement and investigative agencies, including IBAMA, the Chico Mendes Institute for Biodiversity Conservation (ICMBio), and the Federal Police (PF), promoting greater efficiency and reliability in forensic reports and criminal accountability processes. Considering the context in which wildlife trafficking has intensified in virtual environments, particularly following the COVID-19 pandemic, another relevant proposal is the integration of biobanks with artificial intelligence (AI) [31, 32]. This technology enables automated cross-referencing of genetic and morphometric data with existing databases, both within Brazil and internationally, thereby facilitating faster and more accurate pattern recognition and supporting the unraveling of complex trafficking networks and animal modeling studies [32, 33]. AI is also an alternative to conventional analysis methods, which often rely on fragmented information and require manual review. The integration of AI in veterinary anatomy, the use of animal models in preclinical research, and the applications and challenges of ChatGPT in anatomical education were investigated in three studies. In the context of a biobank, the applicability of these tools lies mainly in the management, curation, advanced analysis, and security of the vast volume of data and biological materials. The biobank acts as a structured and secure repository for biological materials and extensive, high-quality datasets (including images, omics data, and animal model results), which are the essential raw materials for the AI applications in diagnostics, R&D, and research [32–34]. Another topic that must be mentioned is the importance of biobanks in One Health, which can be extremely useful, especially when the information is crossed and linked, which could also be performed by AI. Through all biobank information, the AI could predict which pathogens could be emerging. The biobank plays a central role in the One Health framework by integrating human, animal, and environmental health through the systematic collection and sharing of biological samples and data. This integration enables cross-disciplinary research on disease origins, including zoonotic transmission, accelerates vaccine and drug development, enhances understanding of the role of biodiversity in health and disease, supports advances in reproductive medicine, and facilitates environmental influence monitoring on health outcomes. Achieving these goals requires effective collaboration across sectors, adoption of standardized protocols, and ethical data sharing practices [35]. The implementation of biobanks aimed at the conservation, monitoring, and combat of wildlife trafficking must adopt a multidisciplinary and interinstitutional approach. In this context, universities and research centers play a fundamental role not only in producing technical-scientific knowledge and developing new technologies but also in training qualified professionals capable of operating across all stages of this network, from academic research to routine application. Coordination among academic institutions, environmental agencies, and public security bodies is crucial to these initiatives’ success (Figure 7).

## CONCLUSION

This systematic review synthesized 15 eligible studies from 2015 to 2023, revealing a progressive maturation in the application of biobanks within veterinary forensic medicine, particularly for addressing wildlife trafficking. Initial publications (2015–2018) emphasized molecular techniques, such as DNA barcoding, for species identification and geographic origin tracing in seized products, including mislabeled fishery items, pangolin scales, and counterfeit mammalian claws. Subsequent research (2020–2023) positioned biobanks as integrative tools, facilitating genetic resource preservation, chain-of-custody documentation, and interdisciplinary linkages among conservation, judicial, and public health domains. Notable advancements included minimally invasive sample sourcing (e.g., feather follicle cells for avians) and cryopreservation protocols for reptiles and amphibians, underscoring biobanks’ utility in enhancing evidentiary reliability and reducing unrecorded trafficking incidents, estimated at approximately 70% globally due to identification deficiencies.

The findings advocate for the operationalization of biobanks as frontline assets in wildlife crime enforcement. In high biodiversity contexts like Brazil, integration with CETAS could enable regionalized sample collection, fostering national databases for rapid species verification and trafficking route mapping [30]. Artificial intelligence-assisted data curation promises accelerated pattern recognition in virtual trafficking networks, post-COVID-19 escalation. Within a One Health paradigm, interconnected biobanks could predict emerging zoonotic threats through cross-referenced datasets, informing policy formulation and interagency collaboration among environmental authorities, veterinarians, and law enforcement. These implications extend to global scales, promoting standardized protocols to bolster judicial admissibility and conservation efficacy.

This review consolidates fragmented evidence on an underexplored topic, elevating the visibility of biobanks’ forensic potential amid rising wildlife crime. Its adherence to Preferred Reporting Items for Systematic Reviews and Meta-Analyses (PRISMA 2020) guidelines ensures methodological rigor, while the narrative synthesis across diverse taxa and regions provides a comprehensive foundation for translational interventions. The inclusion of practical proposals, such as AI integration and multidisciplinary networks, enhances applicability for stakeholders in academia, policy, and practice.

The analysis is constrained by the limited volume of publications (only 15 studies post-2015), reflecting the nascent state of veterinary forensic biobanking and potential publication bias toward conservation over forensic themes. Fragmented data connectivity and heterogeneous methodologies across studies impeded quantitative meta-analysis, relying instead on qualitative synthesis. Geographic skew toward Brazil and select high-trafficking regions may limit generalizability, while the exclusion of non-English/Portuguese literature and gray sources could overlook emerging global initiatives.

Prospective research should prioritize the development of harmonized international protocols for sample traceability, chain-of-custody, and data interoperability, potentially through CITES-aligned frameworks. Longitudinal studies evaluating biobank efficacy in real-world trafficking prosecutions, alongside pilot implementations of artificial intelligence-enhanced platforms, are warranted. Expanding taxonomic coverage to under-represented groups (e.g., invertebrates) and integrating in vivo and in vitro validation of cryopreservation techniques could further advance forensic applications. Collaborative registries of global biobanks would facilitate knowledge sharing, addressing awareness gaps among practitioners.

Biobanks represent a transformative yet underutilized resource in veterinary forensic medicine, bridging conservation imperatives with enforcement needs to combat wildlife trafficking effectively. By overcoming current barriers through standardization and innovation, these repositories hold promise for safeguarding biodiversity, mitigating zoonotic risks, and upholding justice in an era of escalating environmental threats. This review underscores the urgency of concerted multidisciplinary efforts to realize their full potential.

## DATA AVAILABILITY

The supplementary data can be made available from the corresponding author upon request.

## THE DECLARATION OF GENERATIVE AI IN SCIENTIFIC WRITING

To support data synthesis and streamline manuscript preparation, ChatGPT 5.2, NotebookLM, NAPKIN AI, and Microsoft Excel were used to summarize the literature and organize structured content. All AI-assisted output was critically reviewed, edited, and verified by the authors to ensure accuracy, originality, and alignment with the scientific context. This usage complies with the journal authorship and ethical guidelines. No AI tool was used for final data interpretation or authorial decision-making.

## AUTHORS’ CONTRIBUTIONS

NFS, TFS, LGS, FFAV, JKLFN, NYYS, BPR, MCG, FBCM, and NSR: Performed the data acquisition and analysis. NFS, TFS, LGS, FFAV, JKLFN, NYYS, BPR, and MCG: Wrote the manuscript. FBCM and NSR: Reviewed the manuscript. NSR: Supervised the study and guided the data extraction and analysis. All authors have read and approved the final version of the manuscript.
